# Special Issue “Molecular Mechanism in Epithelial-Mesenchymal Transition (EMT) and Fibrosis”

**DOI:** 10.3390/ijms25073808

**Published:** 2024-03-29

**Authors:** Margherita Sisto, Sabrina Lisi

**Affiliations:** Department of Translational Biomedicine and Neuroscience (DiBraiN), Section of Human Anatomy and Histology, University of Bari “Aldo Moro”, Piazza Giulio Cesare 1, I-70124 Bari, Italy

The process known as epithelial-mesenchymal transition (EMT), fundamental for accurate development during embryogenesis, is involved in several pathological mechanisms, such as severe fibrosis and cancer. EMT is a biological process that allows epithelial cells to acquire a mesenchymal morphology with increased migratory activity, invasiveness, elevated resistance to apoptosis, stem-like characteristics, and the increased accumulation of ECM components. During the EMT event, distinct molecular processes are triggered: the loss of junctions and apical–basal polarity by epithelial cells, the induction of transcription factors, the downregulation of epithelial markers and up-regulation of mesenchymal proteins, and the rearrangement of cytoskeletal factors. EMT is linked to wound healing, tissue regeneration, and organ fibrosis. During the course of fibrosis, EMT can lead to organ injury following the constant release of several inflammatory signals. EMT is now considered a meeting point among inflammation, fibrotic conditions, tumors, and diseases characterized by a chronic inflammatory state, such as autoimmune pathologies. However, in spite of numerous findings elaborated in recent years, relatively little is known about the mechanisms involved in fibrosis pathogenesis, how all of these events are combined and participate in the same process, and how the mesenchymal state is conserved. Intensive knowledge of these mechanisms will help to understand the plasticity of this process to invert the metastatic phenotype of many tumors and identify potential therapeutic targets.

This special issue aimed to create a forum to highlight novel molecular mechanisms in EMT linked to fibrosis and provide readers with a broad overview of the current and emerging research in this field. As a result, this issue has been enriched with an outstanding fifteen original articles (five literature reviews and ten original research articles) with diverse research concepts, nevertheless keeping the ideas aligned with the topic.

One of the hot topics of the last year has been the discovery of a molecular link between epithelial to mesenchymal plasticity (EMP), in particular EMT, and organ fibrosis, which has led to the identification of complex molecular mechanisms closely interconnected with each other, which could clarify EMT-dependent fibrosis. Sisto and Lisi have summarized recent data on epigenetic mechanisms implicated in the fibrotic process, with a focus on epigenetic regulation of EMP/EMT-dependent fibrosis. These epigenetic changes have contributed to obtaining more advice on the fibrotic process, and this could represent a promising pathway forward for the identification of innovative therapeutic targets for fibrogenesis.

Among the literature reviews, Yoshie et al. beautifully discuss the role of how EMT is strongly linked to the activation or inactivation of Cl^−^ channels and whether Cl^−^ channels provide clues for explaining the molecular mechanisms of EMT in the fibrotic evolution of asthmatic airway remodeling.

Mottais et al. made an insightful review focusing on EMT molecular mechanisms involved in chronic airway diseases that lead to structural modifications due to altered and excessive profibrotic tissue with progressive organ injury and lung function impairment.

Datlibagi et al. have highlighted recent data about the role of the EMT process implicated in the pathogenesis of proliferative vitreoretinal diseases (PVDs) characterized by progressive fibrotic tissue in the retinal pigmental epithelium. Therefore, this review offers a comprehensive overview of the benefits, advantages, and limitations of the actual models available to analyze EMT in PVDs.

Another review dealing with the role of the EMT as a link between inflammation and lung cancer is the one provided by Odarenko and colleagues. This review presents attractive insights into the analysis of information on the relationship between the inflammatory tumor microenvironment and EMT process and may provide ideas for novel treatment approaches for lung cancer. Furthermore, the discovery of the EMT inhibitors based on pentacyclic triterpenoids (PTs), described in the second part of the paper, decreases the metastatic evolution and stemness of cancer cells, making PTs promising candidates for pulmonary cancer therapy.

An important aspect of idiopathic pulmonary fibrosis (IPF) is the involvement of the numerous cell types identified as contributors to pulmonary fibrosis, and among these, fibroblasts play a central role in the process of fibrogenesis. The identification of these pathogenic fibroblast subsets was investigated by Hou et al. through a meticulous analysis of scRNA-seq data obtained from lung tissues derived from normal and IPF patients. The authors were able to classify fibroblast populations and reveal the molecular and biological characteristics of fibroblast subtypes in fibrotic lung tissue. Moreover, using high-dimensional transcriptomics data analysis, the authors have identified the co-expressed gene modules linked to IPF-fibroblasts and constructed a model specifically focused on IPF-fibroblasts that can be utilized to assess the disease prognosis in IPF patients. These findings have the potential to improve disease prediction and facilitate targeted interventions for patients with IPF.

In the context of the possibility of finding and developing effective drug target selection for Sjögren’s syndrome (SS), most interestingly, Mougeot et al. have explored the effects of epidermal growth factor (EGF) and interferons (IFNs) on the signal transducer and activator of transcription STAT1 and STAT4 at both gene and protein levels in SS cell culture models. They have demonstrated differences in responses to EGF and IFNs regarding both gene and protein levels of STAT1 and STAT4 between salivary gland epithelial cells (SGEC) derived from patients affected by *sicca* and SS. Furthermore, various genetic polymorphisms in the STAT1-STAT4 gene located on chromosome 2 have been confirmed for their link to SS. These results might contribute to understanding the inefficacy of drugs used for SS due to heterogeneity in SS disease manifestations and discovering efficacy potential compounds.

A number of articles studied the effects of different compounds on the EMT process as an antifibrotic strategy. Among them, the study by Chin-Chuan Chen and co-workers has investigated the therapeutic effects of corylin, a flavonoid extracted from *Psoralea corylifolia* L. (Fabaceae), in the context of liver fibrosis. Strikingly, they have demonstrated anti-inflammatory activity of corylin through inhibition of the expression of interleukin (IL)-1β, IL-6, and TNF alpha in the human monocyte cell line THP-1. Furthermore, corylin inhibited the expression of growth arrest-specific gene 6 in human hepatic stellate cells (HSCs) and the activation of the downstream phosphoinositide 3-kinase/protein kinase B pathway. These findings revealed that corylin has elevated anti-inflammatory activity and, thus, could be a potential adjuvant treatment for liver fibrosis.

On this same issue, the article by Marta Kinga Lemieszek et al. describes the therapeutic potential of cathelicidin, an antimicrobial peptide, on the EMT process occurring during lung fibrosis development in the course of hypersensitivity pneumonitis (HP). The drug was tested in a murine model of HP, wherein lung fibrosis was induced by chronic exposure to the extract of *Pantoea agglomerans*. Cathelicidin determines a reduction in the expression of EMT-associated factors such as Snail1, TGFβ1, ZEB1, ZEB2 suggesting the possibility of its use in the prevention and treatment of pulmonary fibrosis.

In the last few years, it has been demonstrated that pathogenic mutations can trigger severe fibrotic diseases. In this context, Klay et al. have conducted a study that is particularly interesting because they have quantified the RNA expression of mutations in telomere-related genes and in surfactant-related genes in patients with sporadic pulmonary fibrosis. This finding could support the development of therapies targeting fibrosis genes that may be beneficial to all patients affected by pulmonary fibrosis, including those with genetic pulmonary fibrosis.

An attractive topic that regards peritoneal sclerosis induced by peritoneal dialysis, a renal replacement therapy for renal failure that induces inflammation and fibrosis in the peritoneum, was investigated by Kunitatsu et al. They have demonstrated peritoneal sclerosis in the livers of rats after the administration of bleomycin and lansoprazole. To understand the involved mechanism, they have analyzed histological changes in the peritoneal tissue around rat livers. The administration of both drugs together, but not individually, thickened the peritoneal tissue around the liver, determining the accumulation of collagen fibers, induced the migration of macrophages and eosinophils, and triggered fibrosis associated with the possible activation of fibroblasts and the possible promotion of the mesothelial-mesenchymal transition.

Li et al. contributed an original study about the fibrotic process resulting from glaucoma filtration surgery (GFS). In this study, an increase in p-STAT3 was observed in activated human tenon fibroblasts (HTFs), which are key cells in subconjunctival fibrosis and are typically used to study fibrosis associated with GFS in vitro. Inhibiting STAT3 in cultured HTFs by pharmacological inactivation reversed the fibrotic responses, such as fibroblast migration, the differentiation of resting fibroblasts into myofibroblasts, and the deposition of ECM, mediated by IL-6 and TGF-β1. In this study, the decrease of the expression of suppressor of cytokine signalling 3 (SOCS3) was evaluated in HTFs treated with IL-6 and TGF-β1, and SOCS3 overexpression rescued ECM accumulation, α-SMA expression, and migration in IL-6- and TGF-β1-induced HTFs by inactivating STAT3. These data suggest that STAT3 plays a key role in the progressive fibrosis induced by different profibrotic pathways and that STAT3 is a potential target for antifibrotic therapies following GFS.

Huiyuan Pang et al. have studied the involvement of the EMT process in preeclampsia (PE), a pregnancy complication beginning after 20 weeks of pregnancy, and investigated the combined effects of 15-PGDH and PGT on the EMT/MET of trophoblasts and decidual stromal cells (DSCs). The authors have demonstrated that placental development and decidualization both involve EMT/MET. Moreover, 15-PGDH expression was downregulated in the placentas but upregulated in the deciduas of PE patients. Inhibiting 15-PGDH promotes a shift to a mesenchymal pattern of trophoblasts and DSCs. In conclusion, these findings may provide a new and alternative therapy for the treatment of PE.

On a different topic, Regina Komsa-Penkova et al. focused their study on the use of adipose tissue-derived MSCs (ADMSCs), which are cellular models that possess the characteristic multi-potency, making them very suitable for tissue engineering applications. In this study, the authors provide morphological and morphometric evidence for the altered mechano-transduction of ADMSCs adhering to oxidized collagen involving both focal adhesions (FA) and YAP/TAZ signaling pathways, aiming to better understand the stem cells’ behaviour in conditions of acute oxidative stress and fibrotic events.

Finally, Pablo Sacristán-Gómez et al. have evaluated a set of EMT markers (E-cadherin, vimentin, α-SMA, and fibronectin) in thyroid tissues derived from patients affected by autoimmune thyroid diseases (AITD). Since that TGF-β contributes to fibrosis and/or transition to mesenchymal phenotypes, an in vitro TGF-β–stimulation assay in a human thyroid cell line was performed to assess the EMT process. They found an increased expression of the mesenchymal markers α-SMA and fibronectin in TFCs in the thyroid glands of patients affected by AITD. The TGF-β-stimulation assay showed an increase in EMT markers, including vimentin, α-SMA, and fibronectin, in thyroid cells that contribute to the pathogenesis of these diseases.

In conclusion, this collection of articles shows that EMT is still on the crest of the wave as a potential target for the treatment of various human pathologies to counteract its evolution. The basic research reported in these articles is therefore fundamental for devising new therapeutic approaches.

